# Three-Dimension Epithelial Segmentation in Optical Coherence Tomography of the Oral Cavity Using Deep Learning

**DOI:** 10.3390/cancers16112144

**Published:** 2024-06-05

**Authors:** Chloe Hill, Jeanie Malone, Kelly Liu, Samson Pak-Yan Ng, Calum MacAulay, Catherine Poh, Pierre Lane

**Affiliations:** 1 Department of Integrative Oncology, British Columbia Cancer Research Institute, 675 W 10th Ave., Vancouver, BC V5Z 1L3, Canada; chloe_hill@sfu.ca (C.H.); jmalone@bccrc.ca (J.M.); keliu@bccrc.ca (K.L.); cmacaula@bccrc.ca (C.M.); cpoh@dentistry.ubc.ca (C.P.); 2 School of Engineering Science, Simon Fraser University, 8888 University Drive, Burnaby, BC V5A 1S6, Canada; 3 School of Biomedical Engineering, University of British Columbia, 2222 Health Sciences Mall, Vancouver, BC V6T 1Z3, Canada; 4 Faculty of Dentistry, University of British Columbia, 2199 Wesbrook Mall, Vancouver, BC V6T 1Z3, Canada; samson@dentistry.ubc.ca; 5 Department of Pathology and Laboratory Medicine, University of British Columbia, 2211 Wesbrook Mall, Vancouver, BC V6T 1Z7, Canada

**Keywords:** optical coherence tomography, oral cancer, deep learning, segmentation

## Abstract

**Simple Summary:**

The diagnosis of oral cancer can require multiple biopsies to increase the likelihood of sampling the most pathologic site within a lesion. Optical coherence tomography (OCT) enables the examination of subsurface morphology and has shown potential in biopsy guidance. OCT captures changes in tissue stratification related to depth, topology, and presence of the epithelial-stromal boundary, which are structural biomarkers for pre-invasive and invasive oral cancer. This study presents a neural network pipeline to simplify OCT interpretation by providing information about epithelial depth and stratification through simple en face maps. U-net models were employed to segment the boundaries of the epithelial layer, and supporting convolutional neural networks were used for identification of the imaging field and artifacts. Non-cancerous, precancerous, and cancerous pathologies across the oral cavity were evaluated. The predictions demonstrate as-good-as or better agreement than inter-rater agreement, suggesting strong predictive power.

**Abstract:**

This paper aims to simplify the application of optical coherence tomography (OCT) for the examination of subsurface morphology in the oral cavity and reduce barriers towards the adoption of OCT as a biopsy guidance device. The aim of this work was to develop automated software tools for the simplified analysis of the large volume of data collected during OCT. Imaging and corresponding histopathology were acquired in-clinic using a wide-field endoscopic OCT system. An annotated dataset (*n* = 294 images) from 60 patients (34 male and 26 female) was assembled to train four unique neural networks. A deep learning pipeline was built using convolutional and modified u-net models to detect the imaging field of view (network 1), detect artifacts (network 2), identify the tissue surface (network 3), and identify the presence and location of the epithelial–stromal boundary (network 4). The area under the curve of the image and artifact detection networks was 1.00 and 0.94, respectively. The Dice similarity score for the surface and epithelial–stromal boundary segmentation networks was 0.98 and 0.83, respectively. Deep learning (DL) techniques can identify the location and variations in the epithelial surface and epithelial–stromal boundary in OCT images of the oral mucosa. Segmentation results can be synthesized into accessible en face maps to allow easier visualization of changes.

## 1. Introduction

The early detection and diagnosis of cancer improves patient prognosis and the potential for successful treatment. In the United States, the 5-year survival rate is 86% for localized head and neck cancers but decreases to 69% for regional cancers and to 40% for distant metastatic cancers [1]. Unfortunately, only 28% of head and neck cancers are detected at the localized stage [2]. Screening methods consist primarily of incisional biopsy and histopathologic examination, which is a burden on both the patient and the healthcare system. The most common treatment for oral cancer is surgical resection; this procedure can result in devastating physiological and psychological effects [3,4,5].

The multi-step progression of healthy oral mucosa through grades of dysplasia to cancer is very well understood. Dysplastic changes typically originate in the epithelium, just above the epithelial–stromal boundary [6]. The World Health Organization (WHO) has identified architectural (presence and degree of epithelial stratification) and cytological (cellular atypia) changes as key indicators of dysplastic progression [7]. Malignant lesions are classified by having breached the epithelial–stromal boundary, surpassing the basement membrane that prevents the spread of cancerous cells into connective tissue, and allowing for potential metastasis [8]. The most prevalent invasive tumor is oral squamous cell carcinoma (OSCC), accounting for over 90% of oral tumors [9]. There are several structural biomarkers for invasive cancer accessible through histological staining, however ensuring that a biopsy sample contains the most pathologic tissue is difficult. Multiple biopsies are often taken to reduce false negatives, as the clinical presentation of benign lesions may appear similar to occult lesions [10].

Optical coherence tomography (OCT) is a volumetric imaging technique that generates images through reconstruction of backscattered light from a low coherence source. While the most widespread clinical application of OCT is retinal imaging [11,12], the utility of OCT as an adjunct screening tool for oral cancer has been previously explored [13,14,15,16]. OCT allows for non-invasive in vivo measurement of epithelial thickness and visualization of architectural changes relating to stratification and structure within the oral cavity [17,18,19]. Bridging the resolution-gap between ultrasound and microscopy, OCT has an axial resolution of ~5–10 μm, providing information at the microstructure level that was previously only available through biopsy, as morphological features imaged in OCT are strongly correlated to those observed in histology [20]. The primary limitation of OCT is a shallow imaging depth, generally collecting data up to 2–3 mm into tissue. Yet, this depth is comparable to that of histological data collection, making it an excellent tool for examining changes to near-surface tissue [21]. 

The clinical adoption of oral OCT requires data analysis tools that can provide rapid and reproducible assessment of tissue morphology, as the large volume of data collected during imaging makes manual assessment intractable. This work proposes applying deep learning methodologies for automated assessment of OCT data to extract tissue layer stratification. The intersection of deep learning and image analysis has been documented in OCT applications, with substantial evidence indicating utility in image segmentation [22,23,24,25,26] and image classification [27,28,29] tasks. We build upon previously presented classical methods for the segmentation of oral OCT [30], which were limited in clinical applicability due to poor generalizability and lengthy processing times. There is a gap in the existing literature for the image analysis of oral OCT, where no pathology agnostic, site agnostic, rapid, and repeatable tool exists to identify structures of interest. We posit that this tool should not provide diagnostic suggestions, but instead empower clinical decision making by providing additional data through the easily interpreted visualization of subsurface morphology.

## 2. Materials and Methods

### 2.1. Imaging System

Images were collected using a custom-built swept-source OCT system. The system, described previously [15], employes a 1310 ± 50 nm laser (Axsun Technologies, Billerica, Middlesex County, MA, USA) to achieve ~7 μm axial resolution in tissue. A helical scanning pattern is facilitated using a rotary pullback device, which collects images up to 90 mm in length, thus allowing large tissue sites to be collected in a single scan. A single-mode fiber delivers light to and collects light from the sample; during in vivo imaging, the optical fiber is packaged into a 1.5 mm diameter catheter which is filled with water to provide refractive index matching and reduce friction during rotation. Two catheter sheath holders were developed to allow imaging of various sites in the oral cavity: a modified dental mirror and a modified saliva ejector. 

**Figure 1:**
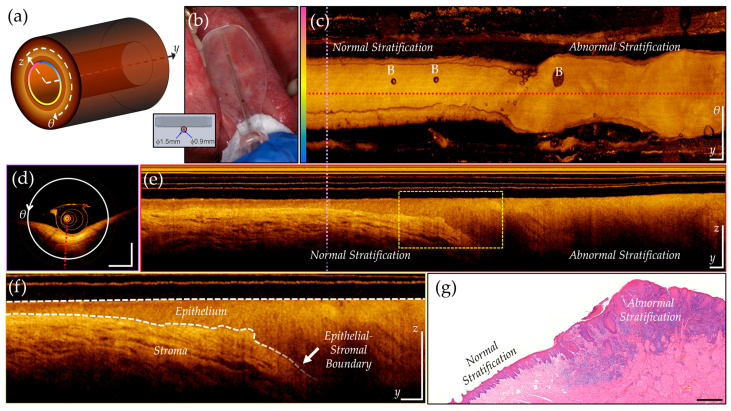
OCT of lateral tongue with biopsy confirmed OSCC. (**a**) Helical scanning coordinate system illustrating z (A-line), θ (azimuthal, pink to blue color gradient), and y (pullback) axes; (**b**) OCT catheter holder based on a modified dental mirror; (**c**) en face mean intensity projection, unwrapped along pink to blue gradient of (**a**), with air bubbles marked by B; (**d**) Cross-sectional view at purple dashed line in (**c**,**e**); (**e**) Longitudinal view at dashed red line in (**c**); (**f**) Inset magnified from yellow dashed box in (**e**), highlighting appearance of epithelial and stromal layers in OCT. Epithelial surface and epithelial–stromal boundary marked by white dashed lines; (**g**) Histology section with regions of normal and abnormal stratification, with corresponding regions marked in (**c**,**e**). Scale bars 1 mm.

Images are acquired in a 3-dimensional cylindrical coordinate system (Figure 1) that includes radial (*z*), azimuthal (*θ*), and pullback (*y*) axes. Unwrapping the volume along the azimuthal axis and applying a mean intensity projection along the radial (A-line) axis creates a 2-dimensional en face projection, shown in Figure 1c. Slicing the volume perpendicular to the pullback axis, at the purple dashed line, provides the cross-section shown in Figure 1d. Slicing perpendicular to the azimuthal axis, at the red dashed line, provides the longitudinal view, shown in Figure 1e. A magnified inset (yellow dashed box) is shown in Figure 1f. The epithelial surface and epithelial–stromal boundary are identified by the white dashed lines, highlighting biomarkers of interest for oral cancer visualized through OCT: epithelial thickening and changes in stratification. Co-registered features can be seen in the histology slice in Figure 1g, depicting regions of normal and abnormal tissue. These features can be observed in the longitudinal image but are challenging to identify in the en face projection. Water-filled catheters sometimes introduce air bubbles into the optical pathway. Air bubbles can be observed in the en face (Figure 1c, indicated with ‘B’), longitudinal and cross section views, and may obscure the tissue underneath.

### 2.2. Data Collection and Assembly 

In vivo imaging of the oral mucosa was approved by the Research Ethics Board of the University of British Columbia and the British Columbia Cancer Agency (H11-02516). Images used in this study were collected from 2014 to 2017. The most pathologic site was identified by an experienced oral medicine specialist (C.P., S.P.-Y.N.) based on clinical presentation using white light illumination, fluorescence visualization, and toluidine blue. One to six OCT scans were performed over the lesion (including some tissue before and after the lesion when possible); a contralateral pullback was also taken when possible. Toluidine blue was applied after OCT to avoid affecting the OCT signal. If deemed clinically necessary, a biopsy was performed after image collection to confirm diagnosis, which could be co-registered to the OCT. 

For this study, 187 volumetric scans were selected from 60 patients (34 male and 26 female). The mean age (M) was 62 years (standard deviation, SD = 14.3). Visual analysis was used to ensure good OCT quality by minimizing the presence of imaging artifacts (≤40% of image). Scans were required to be ≥30 mm in length, with pullback speeds ≤ 10 mm/s and azimuthal scan rates of 100 Hz. Each scan contained 504–512 longitudinal images. One patient was held out of the discovery sets for whole-volume evaluation. Up to three longitudinal images (minimum azimuthal separation of 15°) were selected from the remaining scans for annotation. Images included a variety of sites and pathologies to encourage robustness and generalizability, summarized in Table 1 and Table 2.

### 2.3. Rater Annotations

Supervised deep learning models were used in this study, and, thus, the discovery set required ground truth reference data. Annotations were generated by five raters experienced with analyzing OCT. Manual segmentation of the (1) epithelial surface, (2) epithelial-stromal boundary, and (3) imaging artifacts was completed using in-house annotation software. Artifacts such as air bubbles cause a decrease or complete loss of image intensity and may confound other processing steps. Each image was annotated by three randomly assigned raters. Data was anonymized prior to distribution, blinding raters to identifiers and disease status. A single rater combined these impressions to generate a single reference annotation for each image. Images with high inter-rater disagreement were discussed and a consensus annotation was created in a panel review session.

### 2.4. Pipeline Architecture

A three-step automated pipeline was built to pre-process the volumetric scans, generate predictions via four deep learning networks, post-process the predictions, and synthesize them for visual analysis. The second step of the pipeline employed the following networks:

1. Field-of-view (FOV) detection. A convolutional neural network (CNN) was used to predict either complete tissue contact or partial/no tissue contact for each longitudinal image.
2. Artifact detection. A CNN was used to classify any regions that contained artifacts (bubbles, sheath markers) that might lead to incorrect segmentation predictions.
3. Surface segmentation. A shallow u-net was used to segment the (epithelial) tissue surface.
4. Epithelial–stromal boundary segmentation. A shallow u-net was used to segment the epithelial–stromal boundary.

Post-processing steps are unique to the individual tasks and described below. Predictions are presented as thickness maps which provide information about changes to epithelial thickening, stratification, and the presence of imaging artifacts. Pre- and post-processing methods were completed in MATLAB R2021a. Networks were implemented with PyTorch framework using NVIDIA Cuda v11.4 and Python 3.6.9. All experiments were performed on a Windows 10 operating system, with CPU Intel Core i7-4770 3.40 GHz, GPU NVIDIA GeForce GTX 1660, and 32 GB of RAM.

### 2.5. Data Preparation and Augmentation

Longitudinal images were averaged in the azimuthal direction (out of plane, 5-pixel average) and resampled for isotropic pixel size (10 μm square in tissue). After resampling, images were 382 pixels (3.82 mm) high and 4000 pixels (4 cm) to 9000 pixels (9 cm) wide, depending on the pullback length. The logarithmic intensity (dB) of each image was scaled between the OCT noise floor and the maximum intensity of the image. Images were partitioned into overlapping square tiles to create uniform, memory-aware input information for model training.

The discovery sets are summarized in Table 3. As the classification task required less contextual information, classification tiles were smaller (128 × 128) to allow faster training. Conversely, segmentation tiles were selected to be the maximum base-2 size (256 × 256) without resampling. The deepest 126 pixels contained no relevant information due to signal attenuation and were discarded from each image. Tiles were overlapped (by half their width) to encourage attention at both the left and right side of the image and increase the amount of data available for training.

In the FOV detection task, longitudinal images were randomly selected from a subset of 9 volumetric scans. Images with no tissue present (no probe-tissue contact) were defined as class 0 while those with tissue present (probe-tissue contact over the entire width of the image) were defined as class 1. Images with partial tissue contact were rejected. Similarly, for the artifact detection task, images with no artifacts were defined as class 0 while those with artifacts were defined as class 1. Training, tuning, and test images for this task were selected from the pool of annotated images. Tiles with small artifacts (<30% of the tile) occurring at the edge of the tile were discarded. Segmentation tasks also used annotated images for training, with annotations being converted to ground truth masks. Prior to tiling, images were cropped to remove any regions that did not have an epithelial surface annotation.

### 2.6. Convolutional Neural Network Architectures

**Figure 2:**
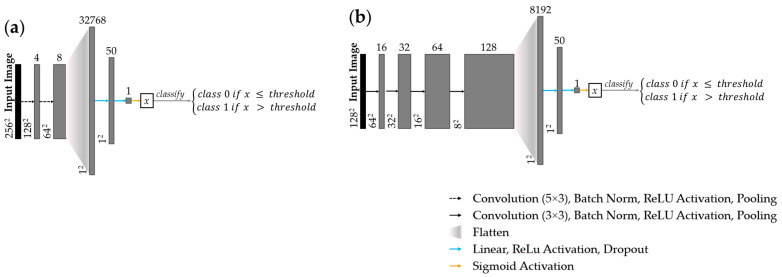
CNN architecture network topology for (**a**) FOV detection task; (**b**) artifact detection task. Arrows represent layer operations (**defined bottom right**); grey boxes represent feature maps, labelled with number of feature maps (**above**) and image dimension (**left**).

A custom CNN was trained for tile classification in the FOV and artifact detection tasks. Shown in Figure 2a, the feature detection layers of the FOV detection network comprised two convolutional layers with ReLU activation and 2 × 2 max pooling. The network was terminated with two dense layers with ReLU activation, and a sigmoid classification layer. Shown in Figure 2b, the artifact detection network followed similar topology but comprised four convolutional layers with ReLU activation and 2 × 2 max pooling.

### 2.7. U-Net Architectures

**Figure 3:**
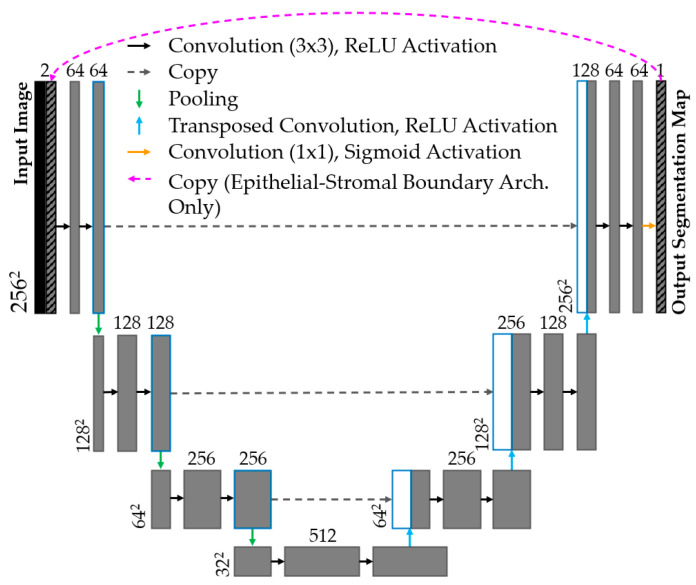
Segmentation u-net topology shown for segmentation tasks. Arrows denote layer operations and grey boxes denote feature maps. Number of feature maps (top) and image dimension (left) are labelled. White boxes are copied feature maps of corresponding blue-outline boxes. Magenta arrow (top) is included in epithelial–stromal boundary architecture only.

Modified u-nets [31] were implemented to identify the epithelial surface and epithelial–stromal boundary. The epithelial surface task used a shallow u-net (encoder: 4 convolutional layers, 3 × 3 kernel, ReLU activation, 2 × 2 max pooling; decoder: 3 convolutional layers, 3 × 3 kernel, ReLU activation, 2 × 2 transposed convolution layers). Shown in Figure 3, the epithelial–stromal boundary task used the same u-net topology with an additional connection, used during training only, concatenating the prediction of the prior epoch to the input of the subsequent epoch.

### 2.8. Model Training

For each task, the discovery set was divided into training, tuning, and test cohorts with approximately 70%, 15%, 15% distribution, ensuring no patient overlap between intra-task cohorts. Stratified sampling was used to promote uniform representation of features of interest across the cohorts. The distribution of features by deep learning task is summarized in Table 4. The epithelial surface task did not have a feature of interest; cohort distribution matched the epithelial–stromal boundary task. 

**Figure 4:**
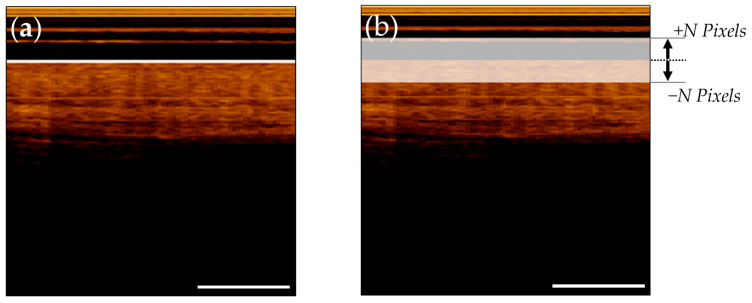
Epithelial surface tile with reference annotation overlaid. (**a**) Raw annotation; (**b**) Thickened annotation. Scale bars 1 mm.

To reduce imbalanced mask content during segmentation tasks, reference annotations were thickened in the vertical (z or A-Line) direction. Various training protocols were explored, evaluating the utility of thickening the segmentation masks. For the epithelial surface task, three models were compared, trained with ±24 pixel masks, ±12 pixel masks, and ±4 pixel masks. The epithelial–stromal boundary task also explored thickened training masks, and analyzed pre-training with coarser masks, and fine-tuning with more precise masks. Figure 4a depicts a raw epithelial surface reference tile overlaid on the image tile. Figure 4b details the training reference tile after thickening (displayed with transparency to allow visualization of the image tile).

### 2.9. Post-Processing

**Figure 5:**
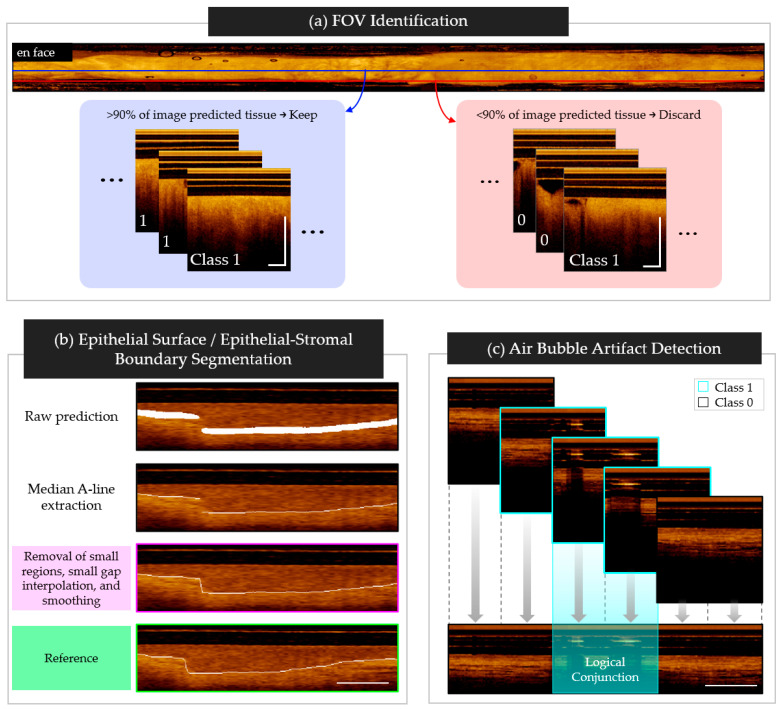
Network post-processing steps. (**a**) Tile aggregation for FOV classification task. Includes example of accepted longitudinal image (left, blue) pulled from center of the en face projection (blue line), and a rejected longitudinal image (right, red) pulled from the tissue-air transition of the en face projection (red line); (**b**) Prediction cleaning for surface segmentation tasks, listed top to bottom: stitched raw prediction; single pixel boundary taken at median A-Line; remove small objects, connect gaps, and smooth prediction (end of process—magenta); reference data (green); (**c**) Tile aggregation for air bubble detection, with overlapping regions classified using logical conjunction. Scale bars 1 mm.

The FOV detection task class predictions were aggregated by original image. To reduce errors arising from incorrect prediction due to imaging artifacts, poor catheter contact, or off-target structures (e.g., gloved hand touching the catheter, mucus), >90% of the image tiles in each longitudinal image were required to be classified as containing tissue (class 1) to continue to subsequent networks. Examples of longitudinal images pulled from the center (approved, blue) and the edge (rejected, red) of the FOV is shown in Figure 5a. 

Post-processing steps for segmentation tasks were developed using model predictions of the test cohort. Steps from the epithelial–stromal boundary task are summarized graphically in Figure 5b, with the final step in magenta, and the reference in green. In both tasks, the raw predictions approximately matched the thickness of the training reference masks. A single pixel thick boundary was created by extracting the median A-Line depth (z-direction) of each tile. Neighboring tiles were then stitched and overlapping regions were averaged. 

For the epithelial surface task, simple morphological operations eliminated small (<10 pixels) regions and subsequently connected any gaps between predictions. Boundaries were smoothed by resampling at every 10th pixel and reconnecting using the p-chip algorithm [32], as shown in Figure 5.

In the epithelial–stromal boundary task, morphological operations were used to (1) connect small gaps (<10 pixels along the pullback (*y*) axis) through linear interpolation, (2) remove small predictions (<25 pixels), (3) connect small gaps (<30 pixels along the pullback axis) through linear interpolation, and (4) smooth predictions through resampling and interpolation with the p-chip algorithm. In steps (1) and (3), regions that were within the pullback-axis limits, but were more than 10-pixels apart along the A-Line depth, were not connected, to best represent the biological organization of oral tissue.

The artifact detection task post-processing steps are summarized in Figure 5c, with class 1 tiles outlined in cyan, and class 0 tiles outlined in black. Classified tiles were stitched into longitudinal images and overlapping tile regions were classified using logical conjunction (i.e., AND), allowing artifact localization within a 64-pixel resolution.

### 2.10. Evaluation Metrics

All metrics were calculated from the test cohort of the discovery set. Balanced accuracy, sensitivity, and specificity are reported for classification tasks, as well as for the receiver operating characteristic (ROC) curve and the precision recall (PR) curve. The area under the ROC curve (AUC) and mean average precision (mAP) are also presented.

For segmentation tasks, the dice similarity coefficient (DSC), percent of positive agreement (PA), percent of negative agreement (NA), and mean A-line error (ME) are reported. The dice similarity score [33],

(1) $$DSC=\frac{2\left|Reference\cap Prediction\right|}{\left|Reference\right|+\left|Prediction\right|},$$

is included for comparability to other segmentation tools; however, this metric demonstrates a bias towards larger objects. Error in comparing small shapes (such as a thin boundaries) is amplified in the DSC, as the small positive sample size is greatly outweighed by the overall sample size (pixel count). Conversely, large shapes may exhibit high DSC despite containing numerous pixels that have been inaccurately segmented [34].

**Figure 6:**
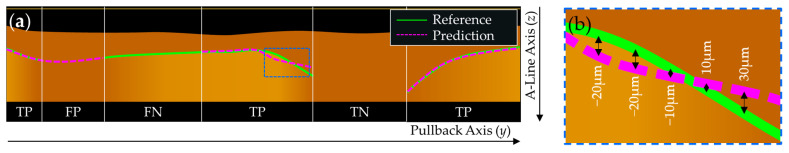
Segmentation metrics. (**a**) Regions of TP, TN, FP, and FN used to calculate PA and NA; (**b**) regions of depth disagreement used to calculate ME, enhanced from blue dashed box in (**a**).

To accommodate this limitation, we also calculate percent of positive and negative agreement,

(2) $$PA=\frac{2TP}{2TP+FP+FN},\;NA=\frac{2TN}{2TN+FP+FN}$$

to represent agreement about boundary existence within each A-line; these metrics are only calculated for the epithelial–stromal boundary task as the epithelial surface post-processing methods guarantee the presence of a boundary. PA and NA are calculated by comparing the annotations for each A-line per tile. For this application, true positive (TP) is defined by boundary presence in the reference and prediction, and true negative (TN) is defined by boundary absence in the reference and prediction. False positive (FP) and false negative (FN) are counted, with the reference used as the ground truth. The ME metric is used to quantify positional (depth) disagreement in each A-line position between predicted versus reference masks; a positive value indicates the prediction is shallower than the reference, while a negative value indicates the opposite. Regions used to calculate PA and NA are shown graphically in Figure 6a. The blue dashed box in Figure 6b demonstrates A-line depth errors used to calculate the ME. Per-image metrics are presented as a single value averaged from all test set predictions.

### 2.11. 3-Dimensional Volumes

Two scans from the excluded patient in the discovery set were analyzed: a grade 2 dysplasia of the lateral tongue, and its contralateral. A total of 504 longitudinal images were generated from each pullback. Each longitudinal image was pre-processed, passed through the four-network pipeline, and post-processed. Predictions were combined to reconstruct a 3-dimensional volume detailing the epithelial layer and imaging artifact. Epithelial thickness maps are corrected for the refractive index of water (n = 1.33), which closely matches that of tissue. A small Gaussian blur was applied to the epithelial thickness maps (10 × 10 pixel kernel) and artifact identification map (5 × 5 pixel kernel) to smooth the edges. Qualitative evaluation is used in the en face view, as manual annotation of complete volumes was not practical. 

## 3. Results

### 3.1. Final Models

For each model, z-score normalization was applied to the discovery set, calculated from the mean and standard deviation of the training cohort. Model weights were initialized per the Kaiming He method [35]. Data augmentation of horizontal flips (50% likelihood) and y-axis shifting (± 10%) was applied to the training cohort. Binary cross entropy [36] and Adam [37] were used as the criterion and optimization functions. Summarized in Table 5, the hyperparameters unique to each task were determined through iterative experimentation.

### 3.2. Convolutional Neural Networks Performance

**Figure 7:**
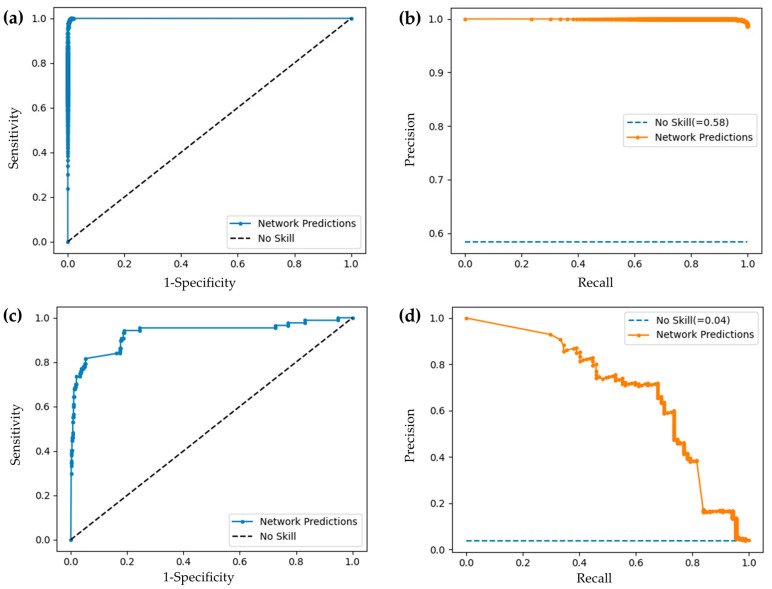
(**a**) ROC and (**b**) PRC curves for FOV classification task; (**c**) ROC and (**d**) PR curves for artifact classification task.

Classification metrics for the FOV and artifact detection models are reported in Table 6; ROC and PR curves are presented in Figure 7, demonstrating improvement over an unskilled model. Evaluation metrics, ROC, and PR curves are calculated from the test set for the respective task.

### 3.3. U-Net Performance

Segmentation metrics are calculated on stitched, post-processed predictions from the test set. DSC values are calculated after thickening the reference and prediction masks; remaining metrics are calculated on the single pixel boundary. 

Three training protocols were evaluated for the epithelial surface segmentation task, exploring the benefit of thickened training masks. The results are detailed in Table 7. With the metrics being nearly equivalent, and acknowledging the bias of the DSC towards larger objects, selection of the smallest pixel error mean and standard deviation indicated the model trained with annotations thickened by ± 12 pixels as superior.

We also explored the effects of thickening the training masks in the epithelial–stromal boundary task, but also analyzed pre-training with coarser masks, and fine-tuning with more precise masks. The results are summarized in Table 8. The affinity of the DSC towards larger objects is again observed. Yet, this protocol performed poorly in other metrics. While protocol 2 presents superior ME metrics, this simpler protocol suffered from the lowest PA and NA values, indicating poor representation of epithelial–stromal boundary detection. Protocol 3 yielded the highest DSC among comparable models, and the highest PA and NA values, indicating the best agreement with regions both with and without an identifiable visible epithelial–stromal boundary. The remaining metrics are similar in protocols 3–5. 

**Figure 8:**
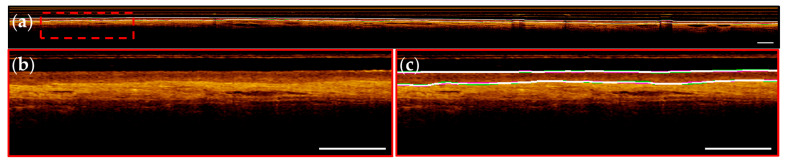
Contralateral ventral tongue. (**a**) Image with reference (green) and prediction (magenta) annotations; white annotations indicate overlap; (**b**) Magnified region of red dashed box in (**a**); (**c**) With annotation overlay (bolded for visualization). Scale bars 1 mm.

**Figure 9:**
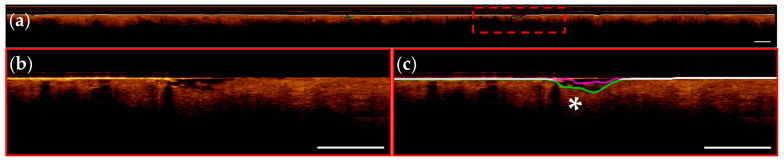
OSCC of the ventral tongue. (**a**) Image with reference (green) and prediction (magenta) annotations; white annotations indicate overlap; (**b**) Magnified region of red dashed box in (**a**); (**c**) With annotation overlay (bolded for visualization), white star indicates region of tissue fold confounding the network. Scale bars 1 mm.

Representative images from patients within the segmentation test set are presented below; images are taken from apparent normal (contralateral, Figure 8) and pathologic (biopsy confirmed OSCC, Figure 9) scans of the ventral tongue. Whole images are presented in Figure 8a/Figure 9a, magnified insets are presented unannotated in Figure 8b/Figure 9b, and annotated in Figure 8c/Figure 9c. The results from the surface segmentation u-net and epithelial–stromal boundary segmentation u-net are included. Reference and prediction annotations are shown in green and magenta, respectively; regions of agreement are highlighted in white. Good agreement between epithelial boundaries can be visualized in the contralateral image (Figure 8). Folds in the tissue surface, which were infrequent in the training set, are shown to confound the surface segmentation model—marked by a white star in Figure 9c. The complete loss of epithelial stratification characteristic of OSCC is captured by the epithelial–stromal boundary segmentation model, an example of a correctly classified true negative region. 

To validate the success of our models, we present our evaluation metrics calculated against the reference annotations, shown in Table 9. Segmentation is a laborious and subjective task, resulting in human raters being prone to error or differences of opinion regarding the boundary location. Model success may be indicated if the reference-prediction metrics are within the distribution of inter-rater metrics. 

### 3.4. Evaluation of 3-Dimensional Volumes 

**Figure 10:**
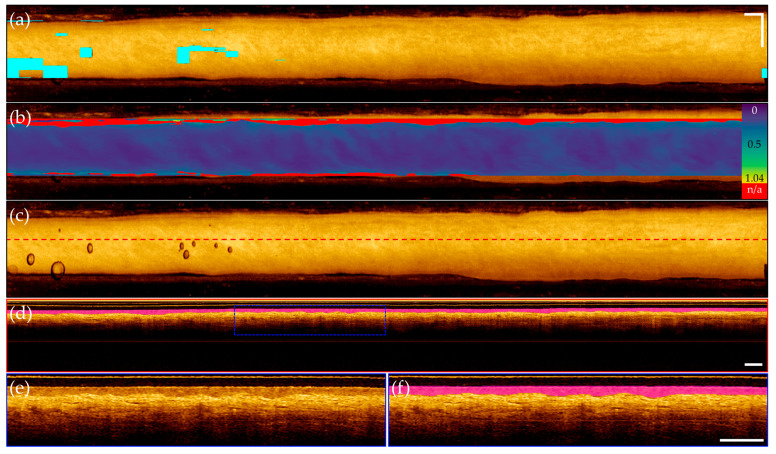
Contralateral pullback of the lateral tongue. (**a**) En face projection with artifact overlay; (**b**) En face projection with epithelial thickness map. Color bar indicates 0 to 1.04 mm thickness, with bright red indicating no visible epithelial-stromal boundary; (**c**) Unannotated en face projection with red dashed line indicating location of longitudinal image in (**d**); (**d**) Longitudinal image with predicted epithelial layer overlay (magenta); (**e**) Inset of blue dashed box in (**d**), unannotated; (**f**) Inset of blue dashed box in (**d**), mirroring inset of (**e**) with annotation. Scale bars 1 mm.

**Figure 11:**
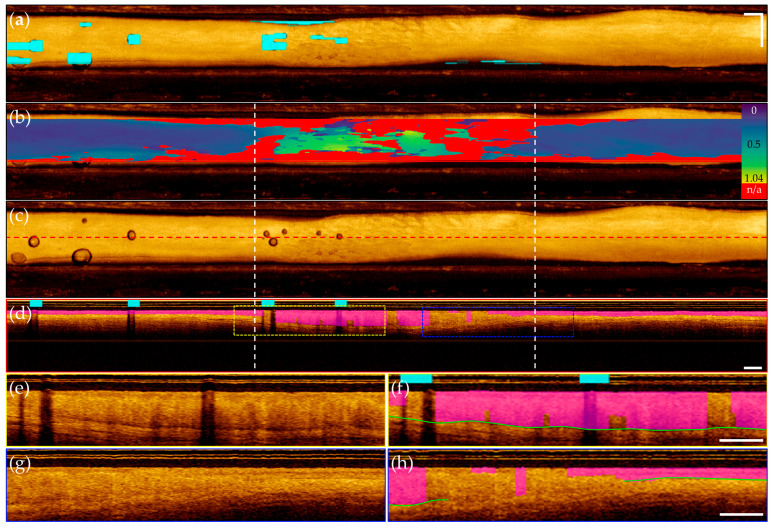
Grade 2 dysplasia of the lateral tongue. (**a**) En face projection with artifact overlay (cyan); (**b**) En face projection with epithelial thickness map. Blue to yellow color bar indicates 0 to 1.04 mm thickness, with red indicating no visible epithelial–stromal boundary. White dashed lines flank predicted region of epithelial thickening and loss, also seen in (**c**,**d**); (**c**) nannotated *en face* projection with red dashed line indicating location of longitudinal image in (**d**); (**d**) Longitudinal image with predicted epithelial layer overlay (magenta) and identified artifact overlay (cyan); (**e**) Inset of yellow dashed box in (**d**), unannotated; (**f**) Inset of yellow dashed box in (**d**) mirroring inset of (**e**) with annotation, green line indicating rater segmentation; (**g**) Inset of blue dashed box in (**d**), unannotated; (**h**) Inset of blue dashed box in (**d**), mirroring inset of (**h**) with annotation, green line indicating rater segmentation. Scale bars 1 mm.

Synthesis of the 3-dimensional prediction data volumes are shown for the contralateral volume (Figure 10) and the dysplastic volume (Figure 11). For each volume, a mask highlighting imaging artifacts is included in Figure 10a/Figure 11a, and in Figure 10b/Figure 11b, an epithelial thickness map, identifying regions of broken or missing epithelial–stromal boundary. Epithelial thickening is demonstrated with the MATLB parula colormap, with darker blue regions indicating thinner epithelium, and yellow regions indicating thickened epithelium. In Figure 10c/Figure 11c, an unannotated en face is included for reference with a red dashed line indicating location of a sample longitudinal image. This image is shown annotated in Figure 10d/Figure 11d with epithelial layer prediction (magenta) and artifact locations (cyan, Figure 11d only). Insets of Figure 10d (blue dashed box) are shown in Figure 10e,f. Insets of Figure 11d (yellow and blue dashed boxes) are included in Figure 11e–h. Annotated panels include a green line identifying rater segmentation for dysplastic insets. 

Both volumes demonstrated accurate localization of the imaging artifacts shown in Figure 10a/Figure 11a, as well as good identification of the FOV, seen in Figure 10b/Figure 11b. Evaluation of the contralateral volume (Figure 10) displays near uniform epithelial thickness in Figure 10b, aligning with the expected biology of the tissue. Precise segmentation of the epithelial surface and epithelial-stromal boundary is observed, supported by isolating a single longitudinal image in Figure 10d, with insets in Figure 10e–f demonstrating accurate epithelial layer identification. Comparatively, evaluation of the dysplastic volume (Figure 11) demonstrates epithelial thickening and loss of the epithelial-stromal boundary characteristic to oral dysplasia in Figure 11b. From the left, the map indicates a slow thickening of the epithelial layer, while the right indicates a sharp transition from stratified into non-stratified tissue. Isolation of a longitudinal image shown in Figure 11d, pulled from the center of the imaging FOV, allows easier visualization of this phenomena. While inconsistencies in the segmentation of the epithelial–stromal boundary are present, with errors highlighted by rater segmentation (green line) in Figure 11f,h, the general trend of abnormal tissue towards the center of the tissue, compared to the more normal stratification tissue towards the image edges, is captured. 

## 4. Discussion 

We present a novel pipeline for the automated segmentation of oral OCT, using deep learning and image processing methods to provide repeatable, time-sensitive information about epithelial status. Our approach is unique as it employs segmentation strategies to characterize the epithelial layer, rather than to classify disease status. This contrasts with prior in vivo oral OCT studies, which were used to triage healthy, pre-cancerous, and cancerous samples [16,38,39] or to classify states of dysplasia [38]. While these methods achieved high agreement, they are constrained by rigid classification labels, and either have limited transparency in the diagnostic process or employ explainability methods to foster physician trust. Instead, we propose a tool to retrieve oral cancer biomarkers and empower clinicians with morphological information upon which to make treatment plan recommendations. 

The processing pipeline employed four independent deep learning networks, preventing the propagation of errors through the analysis pipeline, while simplifying the adaptation (domain-transfer) of these networks to other datasets (lung or cervical OCT, for example). Further investigation into generalizability across oral cavity sites is warranted, where changes in imaging site and pathology may influence network predictions. The networks presented in this research are unavoidably biased towards the more prevalent presentation of oral cancer of the tongue, comprising 71.7% of our data. Whereas the presentation of the epithelial surface (tissue surface) is consistent across imaging site and pathology, the site imbalance in training data may influence network predictions of the epithelial–stromal boundary in sites other than the tongue. 

### 4.1. Convolutional Neural Networks

Training data for the FOV classification task excluded images near the tissue–air transition, as inconsistent contact required the manual labelling of tiles. However, this step of the pipeline is designed to discard entire longitudinal images, and misclassification of individual tiles is mostly managed through averaging during post-processing. Current methods may be too lenient at the edges of tissue contact, for example, at the upper border of Figure 10b. While these images contain oral tissue, the lack of a visible epithelial–stromal boundary is likely not attributed to epithelial thickening, but rather a result of the incident angle of the scanning beam approaching perpendicular to the tissue surface. Consequentially, these images proceed to segmentation steps and are either predicted with thickened epithelium, or marked as N/A. Another potential limitation of this approach includes an intolerance for scans experiencing non-uniform rotational distortion, an occasional artifact due to the helical scanning endoscopic OCT system [40]. However, this effect occurs primarily when the imaging catheter movement is restricted during collection, which is rare when collecting oral OCT due to the accessibility of the site of interest. 

Training data for the artifact classification task excluded image tiles that featured small, borderline artifacts. Potential errors arising from under-representing this type of image are accommodated through overlapping tiles. Stitching tiles using logical conjunction allows for more focal identification of the regions of artifact. However, as observed in Figure 10a and Figure 11a, applying classification labels to whole tiles results in over-identification of artifact regions; a better suited method may be segmentation of the en face projection. If the artifacts of most concern are air bubbles, en face projections could be examined over a reduced A-Line depth to readily identify the sheath and holder region. This would reduce pre-processing time.

### 4.2. U-Nets

U-net models were trained on thickened masks to reduce class imbalance. Size bias is apparent in the DSC metrics of the thicker training masks, yet limitations of these training protocols can be observed in the other metrics. Selection of the ±12 pixel thick mask for the epithelial surface task allowed the necessary precision to avoid basing predictions on the imaging core artifacts, but provided sufficient spatial information that remained useable at the lowest levels of the network. For the more complex epithelial–stromal boundary segmentation task, fine-tuning with thinner masks generated improvements in protocols 3 and 5. This difference may be attributed to the more challenging task, or the adaptation of the network topology, where the input layer contained both the input image tile, and the prediction of the previous epoch. Training protocol 3 exhibited the best response to post-processing methods, despite the algorithm being developed using the predictions of protocol 5. 

The uniqueness of this approach limits its comparability with prior studies as, to our knowledge, no other deep learning segmentation applications have been applied to oral OCT. Evaluation metrics calculated against the reference annotations were presented in Table 9. Comparison of these results to reference prediction metrics for the epithelial surface task and the epithelial–stromal boundary task demonstrates that deep learning methods offer an improvement over manual annotation metric. The A-Line resolution of these images is 10 μm; thus, errors beyond this resolution indicate high agreement. Some poor inter-rater agreement may be attributed to labelling errors rather than misidentification of the surface of interest: this type of error supports the motivation for automated methods. 

Challenges identified in the epithelial surface segmentation task include a limited selection of images containing unique features: longitudinal images where unknown materials occlude the image (*n* = 3) and tissue folds occurring in softer mucosal membranes (*n* = 2). An example of this can be seen in Figure 9c, denoted by a white star. Generally, models struggled to decipher irregular substances (e.g., mucous, keratinization) or structures (e.g., tissue folds and gaps) resulting in incorrect predictions. This error may be addressable with increased sample size including more cases in the training data. 

It is evident that segmentation of the epithelial–stromal boundary is a more complex task for both manual and automated methods, in comparison to the epithelial surface task. The loss of stratification characteristic of cancerous and pre-cancerous oral lesions is difficult to quantify using manual annotations, and some cases required the raters to use contextual information present in the entire longitudinal slice. Accordingly, the image tiling step introduces a network limitation as this context is lost. 

### 4.3. Comparison of Predictions for Contralateral and Dysplastic OCT Volumes

The distinction between epithelial and stromal layers in the contralateral volume is well captured by model predictions, which is particularly clear in the longitudinal image shown in Figure 10d, with inset comparisons in (e) and (f). The epithelial–stromal boundary was segmented for 91.5% of the in-FOV tissue, with the missing 8.5% occurring at the tissue edges. The average predicted epithelial thickness is 208 μm, in agreement with prior research measuring the epithelial thickness of the lateral tongue using OCT (216 ± 59 μm) [41]. 

Conversely, the delineation between the epithelial and stromal layers becomes more nuanced in the dysplastic case, shown in Figure 11, particularly in the region of epithelial thickening indicated by white dashed lines in (b)–(d). The transition across the layers becomes less sharp and, while normal tissue morphology dictates the epithelial–stromal boundary, is visible across the entire image; the network delivers a discontinuous prediction. We hypothesize a few scenarios that result in incorrect model predictions. As discussed above, the loss of contextual information restricts available data upon which to make segmentation predictions. Moreover, as the epithelial layer thickens, the transitionary zone moves lower in the longitudinal image and approaches the maximum imaging depth of our system. At this depth, the signal-to-noise ratio decreases as the light attenuates towards the level of the noise floor, and the transition between the two layers is more difficult to delineate. In cases with extreme epithelial thickening, the epithelial-stromal boundary cannot be visualized at all. Finally, as oral tissue transitions through pre- and potentially malignant lesions towards cancer, changes in cell size, content, and organization alters the scattering properties [42] of the tissue and OCT images become more homogeneous in appearance, further complicating the epithelial–stromal boundary segmentation task. Continued development of complex architectures may improve such errors. Currently, the epithelial–stromal boundary was segmented for 68.7% of the in-FOV tissue and the average predicted epithelial thickness is 319 μm. Both the loss of visible stratification and increased epithelial thickness agree with the expected changes arising in dysplastic tissue.

This work highlights the opportunity for automated depth analysis of OCT data, wherein differences in tissue content are non-obvious in the en face view, and the quantity of longitudinal images renders manual assessment unmanageable. Additionally, presenting a depth of information in the en face view allows for qualitative image interpretation. We acknowledge this study is limited by the relatively small number of patients (N = 60) and scans (N = 187) that were considered.

## 5. Conclusions

A novel deep learning pipeline is presented as a tool to detect and quantify the presence and depth of the epithelial layer in OCT images, potentially decreasing barriers towards clinical applications of oral OCT. Defining the tissue analysis task to be completed through segmentation allows for a more generalized approach, one not restricted by specific oral sites and diseases. Built with two CNNs, for FOV and artifact detection, and two modified u-nets, for epithelial layer isolation, this pipeline provides fast and reproducible results. The comparison of inter-rater agreement to network predictions demonstrates as-good-as or better agreement, and the evaluation of whole OCT volumes of exemplifies the ability to identify pathological progression. 

Automated tools like the one presented here, for epithelial segmentation and detection of abnormal tissue stratification (loss of the epithelial–stromal boundary), will enable the future development of tools that quantify epithelial features like thickness, attenuation coefficient, and interdigitation of the epithelium and stroma. Robust quantification of these features may offer the potential for automated monitoring of pre- and potentially malignant oral lesions, guiding biopsy site selection, to reduce false negative biopsies, and intra-operative margin assessment during tumor resection procedures.

## Figures and Tables

**Table 1:** Summary of dataset pathology.

Diagnosis	No. Scans	% of Dataset	No. Longitudinal Images	% of Dataset
Contralateral (assumed normal)	59	31.6	133	45.2
Benign (pathology confirmed)	14	7.5	19	6.5
Scar	3	1.6	4	1.4
Trauma	1	0.5	1	0.3
Reactive Fibroma	1	0.5	1	0.3
Actinic Cheilitis	2	1.1	3	1.0
Hyperplasia	2	1.1	2	0.7
Verrucous Hyperplasia	3	1.6	4	1.4
Dysplasia Grade 1	24	12.8	31	10.5
Dysplasia Grade 2	21	11.2	25	8.5
Dysplasia Grade 3	12	6.4	18	6.1
Carcinoma In Situ	5	2.7	7	2.4
Lentigo Maligna	4	2.1	5	1.7
OSCC	27	14.4	32	10.9
Verrucous Carcinoma	9	4.8	9	3.1
Total	187	100.0	294	100.0

Abbreviations: OSCC, oral squamous cell carcinoma.

**Table 2:** Summary of dataset imaging site.

Site	No. Scans	% of Dataset	No. Longitudinal Images	% of Dataset
Buccal Mucosa	23	12.3	38	12.9
Floor Of Mouth	8	4.3	13	4.4
Gingiva	10	5.3	11	3.7
Labial Mucosa	4	2.1	8	2.7
Lower Lip	1	0.5	1	0.3
Tongue—Dorsal	4	2.1	6	2.0
Tongue—Lateral	55	29.4	86	29.3
Tongue—Ventral	76	40.6	124	42.2
Vestibule	6	3.2	7	2.4
Total	187	100.0	294	100.0

**Table 3:** Summary of discovery sets for deep learning tasks.

Task	Model	No. Patients	No. Scans	No. Images	No. Tiles	Tile Size (Overlap) [pixels]
FOV Detection	Classification	9	9	2427	Class 0: 64,250 Class 1: 87,376	256 × 256 (128)
Artifact Detection	Classification	59	864	288	Class 0: 20,467 Class 1: 1459	128 × 128 (64)
Epithelial Surface	Segmentation	59	864	288	11,356	256 × 256 (128)
Epithelial–Stromal Boundary	Segmentation	59	864	288	11,356	256 × 256 (128)

Abbreviations: FOV, field of view.

**Table 4:** Distribution of features by deep learning task.

Task	Feature of Interest	Distribution
FOV Detection	Tissue Contact	In FOV: ~60% Out of FOV: ~40%
Artifact Detection	Presence of Artifact	Present: 7% Absent: 93%
Epithelial Surface	N/A	N/A
Epithelial-Stromal Boundary	Tissue Stratification	Complete: ~70% Broken: ~15% Absent: ~15%

Abbreviations: FOV, field of view. N/A, not applicable.

**Table 5:** Training hyperparameters for model development.

Task	No. of Epochs (Early Stopping Epoch)	Early Stopping Scheme	Batch Size	LR Scheduler
FOV Detection	10 (2)	patience = 5, Δ_min_ = 0.01 mode = min. loss	64	patience = 3, factor = 0.1, min. LR = 1 × 10^−8^
Epithelial Surface	30 (19)	patience = 5, Δ_min_ = 0.01 mode = min. loss	8	patience = 5, factor = 0.1, min. LR = 1 × 10^−8^
Epithelial–Stromal Boundary	20 (9, 8) *	patience = 5, Δ_min_ = 0.01 mode = min. loss	8	patience = 5, factor = 0.1, min. LR = 1 × 10^−8^
Artifact Detection	30 (18)	patience = 5, Δ_min_ = 0.01 mode = min. loss	64	patience = 3, factor = 0.1, min. LR = 1 × 10^−7^

* Number of epochs for pre-training and fine-tuning. Abbreviations: LR, learning rate; FOV, field of view.

**Table 6:** Test Set Metrics for FOV and Artifact Detection CNNs.

Task	No. Tiles	Bal. Accuracy (%)	Sensitivity (%)	Specificity (%)	AUC	mAP
FOV Detection	17,404	100.0 *	100.0 *	100.0 *	1.00	1.00
Artifact Detection	2345	75.5	99.1	52.3	0.94	0.68

* After aggregating tiles. Abbreviations: FOV, field of view; AUC, area under the curve; mAP, mean average precision.

**Table 7:** Test set metrics for epithelial surface training protocols. Best results marked in **bold**.

Training Protocol	DSC	ME (μm) (M ± SD)
±24 pixels	**0.99**	0.4 ± 9.9
±12 pixels *	0.98	**0.4** **± 9.1**
±4 pixels	0.94	−0.9 ± 10.4

* Selected training protocol. Abbreviations: DSC, dice similarity coefficient; ME, mean A-line error; M, mean; SD, standard deviation.

**Table 8:** Test set metrics for epithelial–stromal boundary training protocols. The best results are **bolded**.

Training Protocol	DSC	PA (%)	NA (%)	ME (μm) (M ± SD)
±12 pixels	**0.91**	95.8	80.9	2.9 ± 21.5
2. ±4 pixels	0.76	93.1	75.1	0.1 ± 16.2
3. ±12 → ±4 pixels *	0.83	**95.8**	**81.7**	−0.5 ± 19.3
4. ±24 → ±4 pixels	0.76	95.1	80.1	1.5 ± 20.3
5. ±24 → ±12 → ±4 pixels	0.79	95.3	81.2	**−0.1** **± 19.8**

* Selected training protocol. Abbreviations: DSC, dice similarity coefficient; PA, positive agreement; NA, negative agreement; ME, mean A-line error; M, mean; SD, standard deviation.

**Table 9:** Rater-reference metrics for segmentation networks.

Task	Metric	Model	Rater 1	Rater 2	Rater 3	Rater 4	Rater 5
Epithelium	DSC	0.98	0.96	0.96	0.97	0.97	0.95
	ME (μm)	0.4 ± 9.1	5.4 ± 11.4	2.1 ± 11.2	2.4 ± 11.1	3.6 ± 10.7	7.2 ± 13.5
Epithelial–Stromal Boundary	DSC	0.83	0.78	0.64	0.75	0.75	0.73
	PA (%)	95.8	97.2	89.2	95.1	95.4	87.9
	NA (%)	81.7	85.3	69.1	67.9	74.0	56.8
	ME (μm)	−0.5 ± 19.3	0.6 ± 24.2	2.9 ± 32.8	−3.8 ± 24.8	−1.7 ± 18.5	−0.5 ± 14.7

Abbreviations: DSC, dice similarity coefficient; ME, mean A-line error; PA, positive agreement; NA, negative agreement.

## Data Availability

The raw data supporting the conclusions of this article and the code used to process it will be made available by the authors on request.
